# Emergence of spatial behavioral function and associated mossy fiber connectivity and c-Fos labeling patterns in the hippocampus of rats

**DOI:** 10.12688/f1000research.6822.1

**Published:** 2015-07-27

**Authors:** Rachel Comba, Nicole Gervais, Dave Mumby, Matthew Holahan

**Affiliations:** 1 Department of Neuroscience, Carleton University, Ottawa, ON, K1S 5B6, Canada; 2 Department of Psychology, Concordia University, Montreal, QC, H4B 1R6, Canada

**Keywords:** water maze, object exploration, memory, postnatal

## Abstract

Improvement on spatial tasks is observed during a late, postnatal developmental period (PND18 – PND24).  The purpose of the current work was 1) to determine whether the emergence of spatial-behavioral function was based on the ability to generate appropriate behavioral output; 2) to assess whether mossy fiber connectivity patterns preceded the emergence of spatial-behavioral function; 3) to explore functional changes in the hippocampus to determine whether activity in hippocampal networks occurred in a training-dependent or developmentally-dependent fashion.  To these ends, male, Long Evans rats were trained on a spatial water or dry maze task for one day (PND16, PND18 or PND20) then euthanized.  Training on these 2 tasks with opposing behavioral demands (swimming versus exploration) was hypothesized to control for behavioral topology.  Only at PND20 was there evidence of spatial-behavioral function for both tasks.  Examination of synaptophysin staining in the CA3 region (i.e., mossy fiber projections) revealed enhanced connectivity patterns that preceded the emergence of spatial behavior.  Analysis of c-Fos labeling (functional changes) revealed developmentally-dependent increases in c-Fos positive cells in the dentate gyrus, CA3 and CA1 regions whereas training-dependent increases were noted in the CA3 and CA1 regions for the water-maze trained groups.  Results suggest that changes in mossy fiber connectivity in association with enhanced hippocampal functioning precede the emergence of spatial behavior observed at PND20.  The combination of neuroanatomical and behavioural results confirms the hypothesis that this time represents a sensitive period for hippocampal development and modification and the emergence of spatial/ cognitive function.

## Introduction

One brain region that shows connectivity-based changes during postnatal development is the hippocampus. Neurogenesis in the dentate gyrus (DG) peaks in density between postnatal day 15 (PND15; 7) and PND18 ^12, 18, 77^ and the mossy fibers (MF), forming connections between the DG granule cells and CA3 pyramidal cells, show a late, postnatal remodelling ^9, 24, 25, 32^. With increasing evidence that MFs in the adult rat regulate memory function by remodeling ^33, 34, 57– 60^, it is attractive to hypothesize that improved performance on a number of spatial tasks during development ^3, 13, 16, 20, 41, 65, 66, 71, 73^ may be related to connectivity- or functional-based changes in the network properties of the DG-CA3 region.

The main purpose of the present study was to investigate the relationship between the emergence of spatial-behavioral function and the emergence of the neural substrates – both anatomical and functional – that would support optimal spatial-behavioral function. In this respect, one question related to MF connectivity-based developmental changes is whether these changes occur prior to the emergence of spatial behavior or as a result of spatial behavior. This is an issue as adult MF remodelling can occur within a 24-hour period following spatial water maze training ^33^. Another related question is how the functional aspects of hippocampal neurons change during development and whether this too occurs in response to behavioral-based activity. Similar to the MF question is whether hippocampal network activity precedes or results from the emergence of spatial behavior. To investigate this, analysis of c-Fos positive cells in the hippocampal DG, CA3 and CA1 regions was carried out to examine training-associated hippocampal activity and activity associated with neural development.

A final issue explored in the present study was whether improvements in spatial-behavioral performance reflect enhanced hippocampal structural and network activity or emergence of sensori-motor demands required for task performance ^11, 17, 67, 75^. During development, sensori-motor demands of a task could confound firm conclusions concerning the development of spatial-behavioral function (e.g., 30). To clarify this, rats were trained on the hidden-platform water-maze task (aka, Morris water maze task) or a dry maze version of the novelty-preference paradigm where rats are required to recognize that an object is in a place where there had not previously been an object ^26, 54^ for one day on PND16, PND18 or PND20. In this case, spatial representational requirements were similar for both tasks but behavioral topology requirements were different – swimming versus exploration. Comparing performance on these two tasks was hypothesized to determine whether the emergence of spatial-behavioral function was based on the ability to generate appropriate behavioral output or based on the hippocampal spatial navigation neural substrates.

## Materials and Methods

### Subjects

A total of 51 male Long-Evans rats (LER; Charles River, St. Constant, Quebec, Canada) were used. The day the pups were born was marked as postnatal day 0 (PND0). Pups remained with their dams throughout the duration of the study. Rats were housed in a temperature-controlled vivarium in polycarbonite cages with a 12 hour light-dark cycle. Food and water were provided *ad libitum*. All experiments were conducted in accordance with the Canadian Council on Animal Care (CCAC) guidelines and specific protocols approved by the Carleton University Animal Care Committee and the Concordia University Animal Care and Use Committee (Animal Use Protocol Number P13-10).

### Apparatus

***Water maze.*** The water maze was a white, circular, polypropylene pool measuring 124 cm diameter, 31 cm height and filled with water to a depth of 25 cm. Water temperature averaged 23°C and was rendered opaque by the addition of non-toxic white paint. The platform was made of clear Plexiglas measuring 11 cm diameter and was submerged 2.0 cm below the water surface. Distal visual cues were present on the walls of the room surrounding the maze. Rat movement in the pool was tracked using the HVS Image 2100 Tracking System (version 1/09; HVS Image, Buckingham, UK). The water was skimmed and stirred after each trial and the pool was drained and refilled every day.

***Dry maze.*** The dry maze was 122 cm in diameter with Lexan walls and floor measuring 10.5 cm high, 2 mm thick. The floor of the maze was opaque white and the walls were transparent. The two stimulus objects were plastic turquoise water bottles 20 cm high with the widest circumference measuring approximately 23 cm. A small glass jar (7 cm high, 20 cm circumference) was fastened to the bottom of each object with epoxy. The jar lids were inverted and fastened to the floor to allow the objects to be secured in place by screwing the jars into the lids. Holes were drilled at the center of each quadrant to accommodate the lids and to allow counterbalancing of object positions. When not in use, the holes were covered with a small piece of circular white tape. After each trial, the objects and maze were wiped down with water. At the beginning of each day, the floor of the maze and objects were washed with a 70% alcohol solution and the Lexan walls were cleaned with standard window cleaner.

### Behavioral procedures

***Morris Water Maze (MWM).*** For the Morris water maze (MWM) portion, there were three groups with five rats per group (n = 15). Rats underwent fixed hidden-platform water-maze training consisting of 8 trials with a 1-minute inter-trial interval to locate the hidden platform. Training was carried out on one day only at PND16, PND18 or PND20. Each trial began from a different start point on the perimeter of the pool. If a rat did not reach the platform within 60 seconds, they were guided to it. Rats remained on the platform for 30 seconds before being removed, dried, and placed into a holding cage for an additional 30 seconds. Following the final trial, each rat was dried with a towel and placed in another holding cage on a heating pad in the housing room for 15 to 20 minutes before being returned to its home cage. Rats were euthanized with an overdose of 60 mg/kg sodium pentobarbital 1 hour later followed by decapitation.

***Object in a Novel Location (ONL).*** For the Object in a Novel Location (ONL) portion, there were three groups of seven rats (n = 21). Each group was trained and tested on a single day: PND16, PND18 or PND20. For training, rats were individually placed into the arena and allowed to explore. Two identical objects were placed in adjacent arena quadrants and remained in the same location for the familiarization phase. There were three familiarization periods each lasting 7 minutes carried out once per hour over 3 hours. Between familiarization periods, rats were placed back into their home cage with their cage mates.

One and a half hours after the last familiarization period, rats were tested for 5 minutes. For the test, one of the objects was moved to a different quadrant of the arena. This procedure reflects the conventional object-in-novel-place preference test which takes advantage of a rats spontaneous tendency to explore objects that have changed location within an otherwise stable environment ^19, 26, 27^. When rats display such a preference, it is inferred that they have detected a change in location of the object within the environment. To determine preference during the testing phase, an investigation ratio was calculated during the 5 minute testing phase; the proportion of total object-investigation that was spent investigating the displaced object to the total time investigating both objects (t _displaced_/[t _displaced_ + t _not displaced_]). A rat was considered to be investigating an object when its head was within 4 cm and its nose was within a 45° angle from being perpendicular to the object. Investigation was also considered when a rat was rearing with at least one forepaw making contact with the object with the head oriented upwards but not when climbing on top of the object. Each rat was tested once to ensure that the exploration ratio was based solely on the change in the position of the object from the familiarization to the testing phase. Rats were euthanized with an overdose of 60 mg/kg sodium pentobarbital 1 hour later followed by decapitation.

***Home Cage Controls (HCC).*** Three additional, separate groups of rats were euthanized with an overdose of 60 mg/kg sodium pentobarbital on PND16, PND18, or PND20 (n = 5/time point; 15 total) with no behavioural manipulations (home cage controls; HCC). All brains were processed immunohistochemically as described below.

### Immunohistochemistry

***Tissue preparation.*** Brains were immersion fixed in 4%-paraformaldehyde (Sigma; USA) in 0.1M phosphate-buffered saline (PBS) overnight at 4°C. This solution was replaced with 30% sucrose in 0.1M PBS the following day and brains were stored at 4°C until sectioning. Brains were sectioned through the dorsal hippocampus at 60µm on a Leica CM1900 cryostat (Weztler, Germany). Because learning-associated changes in mossy fiber axonal input to the CA3 region are restricted to the most rostral levels of the dorsal hippocampus ^33, 59^, we focussed our immunohistochemical analyses to this region. Sections were stored in a 0.1% sodium azide solution in 0.1M phosphate buffer (PB) at 4°C.

***Synaptophysin staining.*** Sections were washed for 15 min in a 0.2% Triton-X/0.01 M phosphate-buffered saline (T-PBS) then blocked in a 1x animal free blocker (Vector)/T-PBS solution for 1 h at room temperature. Incubation in the primary antibody (rabbit anti-synaptophysin polyclonal antibody from Chemicon/Millipore (Cat#: AB9272), 1:2500) occurred overnight at room temperature. The following day, sections were washed in T-PBS for 15 minutes followed by a 2 hour incubation in the secondary antibody (1:500 goat anti-rabbit Igg (H+L) polyclonal secondary Alexa Fluor 594 from Molecular Probes; Cat# R37117). Sections were given a final rinse in 0.01 M PBS (pH 7.4) for 15 min then mounted on glass slides and coverslipped with glass coverslips adhered with Fluormount (Sigma).

***c-Fos staining.*** Sections adjacent to those stained for synaptophysin were placed in phosphate-buffered saline with Triton X (T-PBS) for three 5 minute washes. They were then incubated in 0.3% hydrogen peroxide (H _2_O _2_) in T-PBS for 15 minutes followed by three, 5 minute washes in T-PBS. Sections were transferred to 1x animal free blocker (AFB; Vector) in T-PBS for 30 minutes at room temperature. Incubation in the primary antibody (rabbit polyclonal anti-c-Fos from Abcam (Cat# ab53036), 1:5000) occurred overnight at room temperature. The following day, tissue was washed in T-PBS for three, 10 minute washes followed by a 2 hour incubation in the secondary antibody (Biotinylated Goat Anti-Rabbit IgG (H+L) Antibody from Vector Laboratories (Cat# BA-1000), 1:1000). Tissue was washed for three 10 minute washes in T-PBS before being placed into an ABC solution (Vector) for 1 hour. The tissue was rinsed in three 5 minute washes using PBS before being placed into a nickel-enhanced 3,3′-Diaminobenzidine (DAB) solution. Sections were then mounted on glass slides, dehydrated (1 minute in distilled H _2_O; 1 minute in 25% ethanol; 2 minutes in 50% ethanol; 5 minutes in 90% ethanol; 10 minutes in 100% ethanol; 20 minutes in Clearene) and cover slipped.

***Synatophysin quantification.*** Fluorescent images of the CA3 region were captured at 20× magnification using a Retiga-2000R camera (QImaging, BC) and an Olympus BX61 research microscope (Olympus-Canada). Three coronal sections were sampled from the dorsal hippocampus. The anterior boundary was defined as the first section where the upper and lower blades of the granule cell layers were equal in length. The posterior limit was 420 μm from this initial anterior starting location. Three coronal sections were sampled from this anterior-posterior boundary with 120 μm between sections. Synaptophysin staining measurements for each region of interest were taken as averages of the number of puncta from the three coronal levels. The areas of the stratum lucidum (SL) and the stratum oriens (SO) were estimated by outlining the synaptophysin-positive region. Synaptophysin-positive puncta were defined as having an intensity value twice that of the background as measured on the stratum radiatum (SR) of the CA3 region and a size restricted to not greater than 5 µm and not less than 1 µm. Comparisons between groups were made using 1) total puncta in the SL; 2) total puncta in the SO; 3) the ratio of puncta in SO to SL to account for size variations between individual animals. An experimenter who was blind to group assignment carried out all analyses.

***c-Fos quantification.*** Utilizing the Optical Fractionator probe, an estimate of the number of c-Fos positive cells in the dentate gyrus (DG), CA3 and CA1 regions of the dorsal hippocampus was undertaken. Stained sections were visualized using an Olympus BX51 brightfield microscope with a motorized stage (Olympus Canada, Markham, ON) and images captured with an Olympus U-CMAD3 camera. Stereo Investigator (version 11.06.1; MBF Bioscience, Williston, VT) software was used for quantification. The region of interest (DG, CA1 or CA3 of the dorsal hippocampus) for each section was traced digitally at 4× magnification. The anterior boundary was defined as the first section where the upper and lower blades of the granule cell layers were equal in length. The posterior limit was 420 μm from this initial anterior starting location. Based on these limits, the volume of the DG measured 17,892 µm ^3^; CA3 volume was 31,637 µm ^3^; CA1 volume was 12,487 µm ^3^. Because of these volume differences across hippocampal subregions, c-Fos data were expressed as c-Fos+ cells per 100 µm ^3^ to ease visual comparisons across subregions. Cell density (as measured on cresyl violet-stained sections from 3 rats from each age group – PND16, PND18 and PND20) in the DG, CA3 and CA1 regions was not different across the 3 age groups (one-way ANOVA; DG (F(2,6) = 3.29, p = 0.089; CA3 (F(2,6) = 3.60, p = 0.094) and CA1 (F(2,6) = 3.56, p = 0.096). Three coronal sections were sampled from this anterior-posterior limit with 120 μm between sections. Counting parameters were set to a counting frame of 30×30 µm ^2^ and a dissector height of 10 µm between the top and bottom guard zones with average mounted section thickness of 35 μm. c-Fos positive cells were quantified using a 60X magnification lens (oil immersion, NA 1.35) when the uppermost tip of c-Fos positive nuclei were in focus within the counting frame and the dissector height. Stereo Investigator software used planar and depth information for each counted nuclei to calculate the volume for the digitally traced region of interest and provide an estimate of the labeled cells per region of interest (DG, CA1 or CA3).

## Results

### Morris Water Maze (MWM)

**Figure 1.**
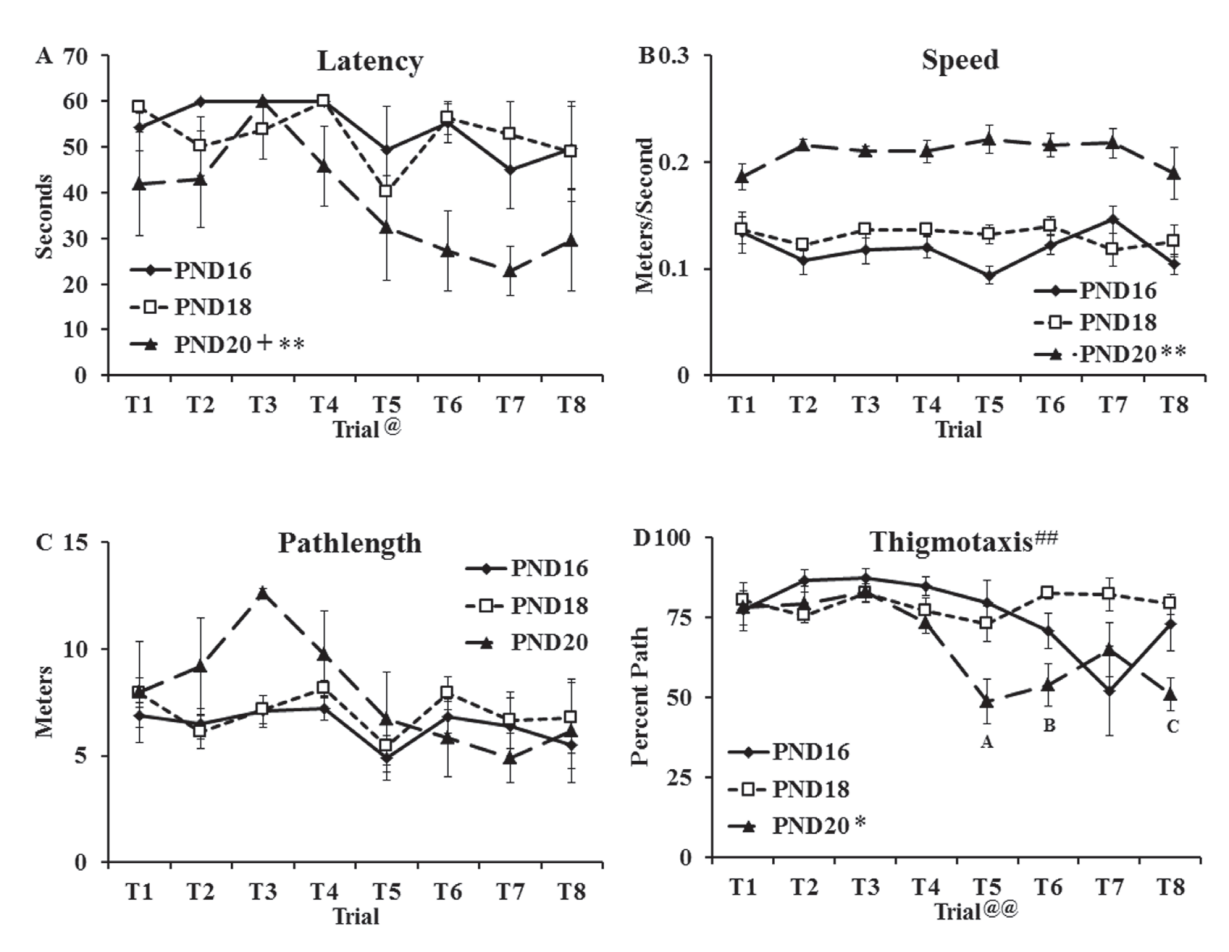
One-day Morris water maze task (MWM). Groups of rats were trained for one day (PND16, PND18, PND20) to locate the hidden platform on the water maze task. Swimming behaviors were captured with HVS Image software (version 1/09) and included: ( **A**) Latency in seconds (mean ± SEM) to reach the platform (60 s cutoff); ( **B**) speed in meters/second (mean ± SEM); ( **C**) pathlength in meters (mean ± SEM); ( **D**) thigmotaxis defined as percent of total pathlength spent within 10 centimeter of the pool wall (mean ± SEM). Significant group differences were found on latency to reach the platform with PND20 group showing shorter latencies than the PND16 (** p < 0.01) and PND18 (+, p < 0.05). Analysis of speed data revealed faster swim speeds in the PND20 group than both other age groups (**, p < 0.01). Measures of thigmotaxis revealed less thigmotaxis in the PND20 group than both other groups (*, p < 0.05). A significant interaction (##, p < 0.01) was also found and specific comparisons showed that the PND20 group spent less time along the edge of the pool on Trials 5 and 8 than PND16 and PND18 ( **A** and **C**; p < 0.05) and on Trial 6 than PND18 ( **B**; p < 0.01). @ and @@ in **A** and **D**, respectively, refer to a main effect of Trial.

 Escape latency ( Figure 1A), speed ( Figure 1B), pathlength ( Figure 1C) and percent pathlength spent swimming along the edge of the pool (thigmotaxis; Figure 1D) data for the water maze task (see Dataset 1) were analyzed using separate two-way, repeated measures ANOVAs (age (PND16, PND18, PND20) as the between factor and trial as the repeated measure). Analysis of latency data ( Figure 1A) revealed main effects of age (F(2,12) = 8.49, p < 0.01) and trial (F(7,14) = 2.44, p < 0.05) but no interaction (F(14,84) < 1.0). Tukey HSD post-hoc tests on the main effect of group revealed that the PND20 group showed shorter latencies to locate the platform than the PND16 group (t(8) = 3.37, p < 0.01) and the PND18 group (t(8) = 3.18, p < 0.05).

Analysis of speed data (meters/sec; Figure 1B) revealed a main effect of age (F(2,12) = 86.18, p < 0.001) but no effect of trial (F(7,14) < 1.0) and no interaction (F(14,84) = 1.43). Tukey HSD post-hoc tests on the main effect of group revealed a significantly faster swim speed in the PND20 group than the PND16 group (t(8) = 13.11, p < 0.001) and PND18 group (t(8) = 9.28, p < 0.001).

Analysis of pathlength data (meters; Figure 1C) revealed no main effect of age (F(2,12) = 2.11, p = 0.163), no effect of trial (F(7,14) = 2.11, p = 0.051) and no interaction (F(14,84) = 1.01, p = 0.45).

Analysis of thigmotactic data (percent of the swimming distance spent within 10 cm of the wall of the pool; Figure 1D) revealed a main effect of age (F(2,12) = 7.07, p < 0.01), a main effect of trial (F(7,14) = 4.92, p < 0.001) and a significant interaction (F(14,84) = 2.97, p < 0.01). Tukey HSD post-hoc tests on the main effect of group revealed significantly less thigmotaxis in the PND20 group than the PND16 group (t(8) = 2.64, p < 0.05) and the PND18 group (t(8) = 3.32, p < 0.05). At Trials 5 and 8, the PND20 group showed less thigmotaxis than the PND16 and PND18 groups (p < 0.05) and at Trial 6, the PND20 group showed less thigmotaxis than the PND18 group (p < 0.01).

Watermaze Dataset Included in this worksheet are the water maze behavioral measures collected for the current work. Measures included latency to locate the platform for each trial, speed on each trial, pathlength to reach the platform for each trial and percent of the pathlength spent close to the perimeter of the pool (thigmotaxis) ^78^. Click here for additional data file. Copyright: © 2015 Comba R et al. 2015 Data associated with the article are available under the terms of the Creative Commons Zero "No rights reserved" data waiver (CC0 1.0 Public domain dedication).

### Object in Novel Location (ONL)

**Figure 2.**
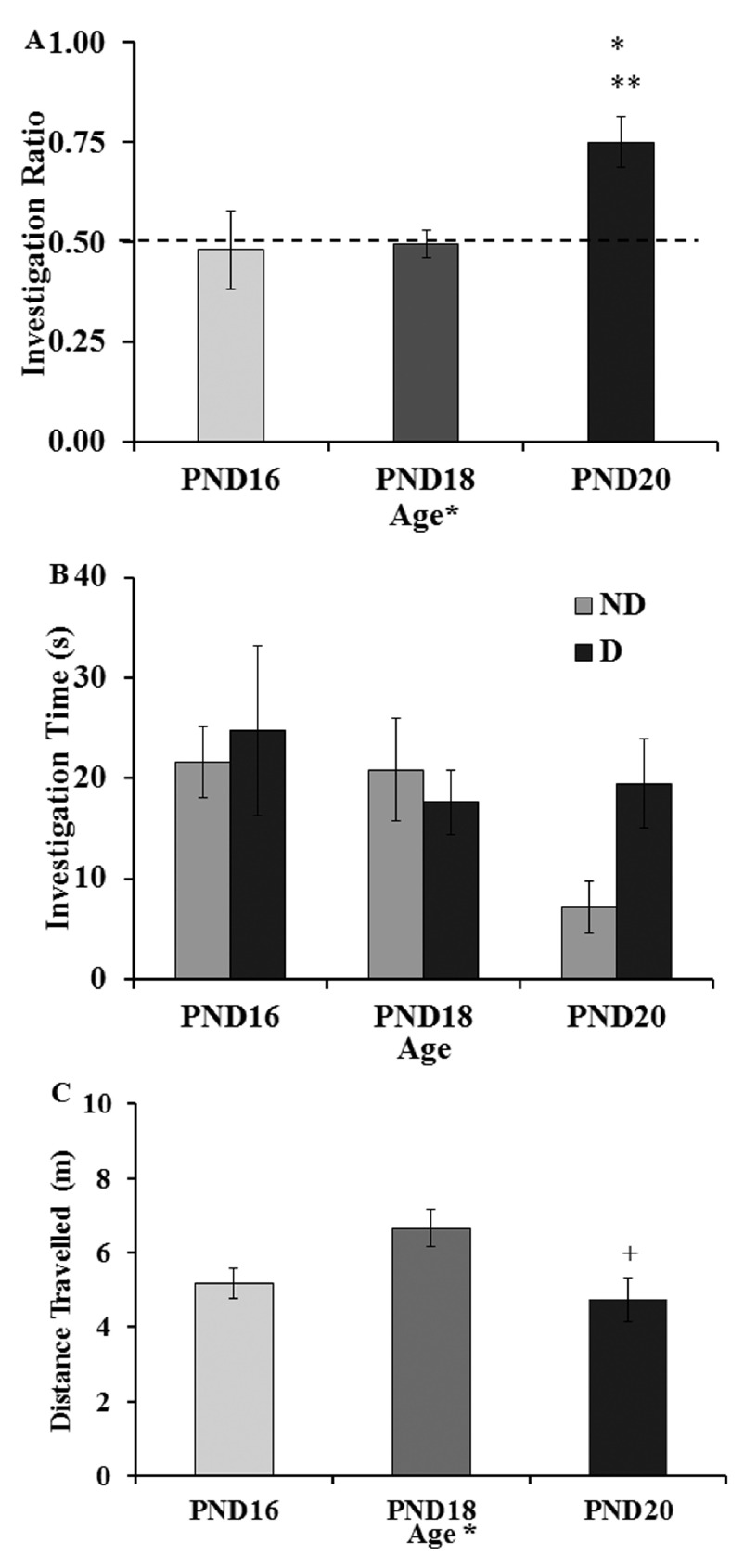
One-day object in a novel location task (ONL). Groups of rats were trained and tested on a single day: PND16, PND18 or PND20. ( **A**) Mean investigation ratios (± SEM) from the entire 5 minute test. Dotted line indicates chance investigation ratios meaning similar times spent investigating the displaced (D) and non-displaced (ND) objects. The PND20 group showed an investigation ratio significantly above chance (** p < 0.01) indicating a preference for the D object over the ND object. The PND20 group also showed a larger investigation ratio than the PND16 and PND18 groups (* p < 0.05). ( **B**) Average total time (seconds ± SEM) spent investigating the D and ND objects. There were no overall age effects or object effects on investigation time. ( **C**) Total distance traveled during the ONL 5 minute test. Data are expressed as average (± SEM) distance in meters. A main effect of age (*, p < 0.05) was found with the PND18 group showing a longer distance traveled than the PND20 group (+, p < 0.05).

The mean investigation ratios (see Dataset 2) for the ONL task are shown in Figure 2A. One-way ANOVA on the investigation ratios between ages revealed a main effect of age (F(2,18) = 4.64, p < 0.05) and Tukey HSD post-hoc tests revealed that the PND20 group showed a larger investigation ratio than both other ages (p < 0.05). The investigation ratios for the PND16 (t(6) = 1.35) and PND18 (t(6) = 0.63) groups were not significantly different from chance (0.5) while the investigation ratio for the PND20 group (t(6) = 2.52, p < 0.05) was significantly greater than chance. An analysis of the times spent investigating the displaced and nondisplaced objects (in seconds; Dataset 2) during the test ( Figure 2B) with a two-way ANOVA (age by investigation time of the displaced and nondisplaced objects) revealed no main effects of age (F(2,18) = 1.60), no main effect of investigation time (F(1,2) = 1.38) and no interaction (F(2,18) = 1.67). One-way ANOVA examining the distance traveled (see Dataset 2) during the test session (in meters; Figure 2C) revealed a main effect age group (F(2,18) = 4.02, p < 0.05). Tukey post-hoc test showed that the PND18 group showed a longer distance traveled during the test than the PND20 group (t(12) = 2.50, p < 0.05). No other age group differences were detected (PND16 vs PND18, t(12) = 1.31).

Drymaze Dataset Included in this worksheet are the data from the object exploration task (dry maze task) consisting of time spent exploring each objected (displaced (d) and nondisplaced (nd)) during the first, second, third fourth and fifth minutes (cumulative time). Distance traveled during the tests are shown below these data ^79^. Click here for additional data file. Copyright: © 2015 Comba R et al. 2015 Data associated with the article are available under the terms of the Creative Commons Zero "No rights reserved" data waiver (CC0 1.0 Public domain dedication).

### Synaptophysin immunohistochemistry

**Figure 3.**
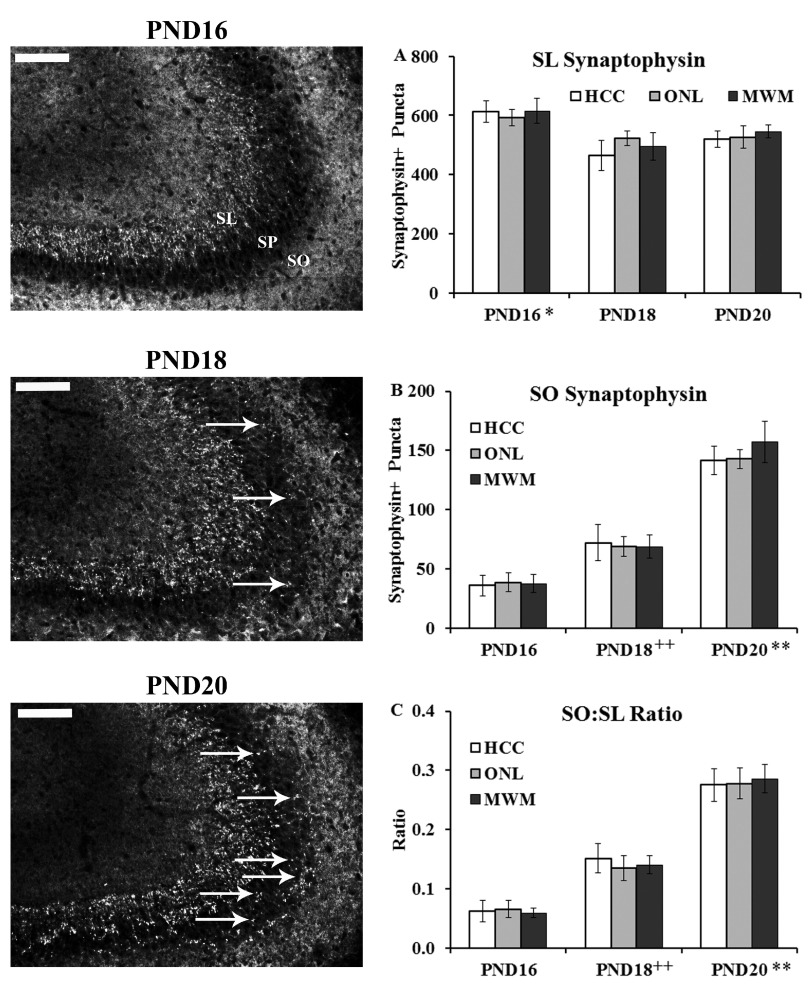
Synaptophysin staining in the CA3 region as an indicator of mossy fiber ( **MF**) projections. Brains from PND16, PND18 and PND20 from home cage control (HCC), object in a novel location (ONL) or Morris water maze (MWM) groups were removed and processed immunohistochemically for synaptophysin staining in the CA3 region as a marker for MF connectivity. Left panels show representative staining from the three age groups (images from MWM condition). Abbreviations: SO: *stratum oriens;* SP: *stratum* pyramisale; SL: *stratum lucidum*. Arrows point to synaptophysin-positive puncta in the SO region. Scale bar = 100 µm. ( **A**) Synaptophysin-positive puncta quantified in the SL region revealed a main effect of age with group PND16 showing more staining than PND18 and PND20 (*, p < 0.05). ( **B**) Synaptophysin-positive puncta quantified in the SO region revealed a main effect of age with group PND18 showing more staining than PND16 (++, p < 0.01) and group PND20 showing more staining than groups PND16 and PND18 (**, p < 0.01). ( **C**) Ratio of synaptophysin-positive puncta quantified in the SO: SL region (to control for potential size variation) revealed a main effect of age with group PND18 showing more staining than PND16 (++, p < 0.01) and group PND20 showing more staining then groups PND16 and PND18 (**, p < 0.01).

**Figure S1.**
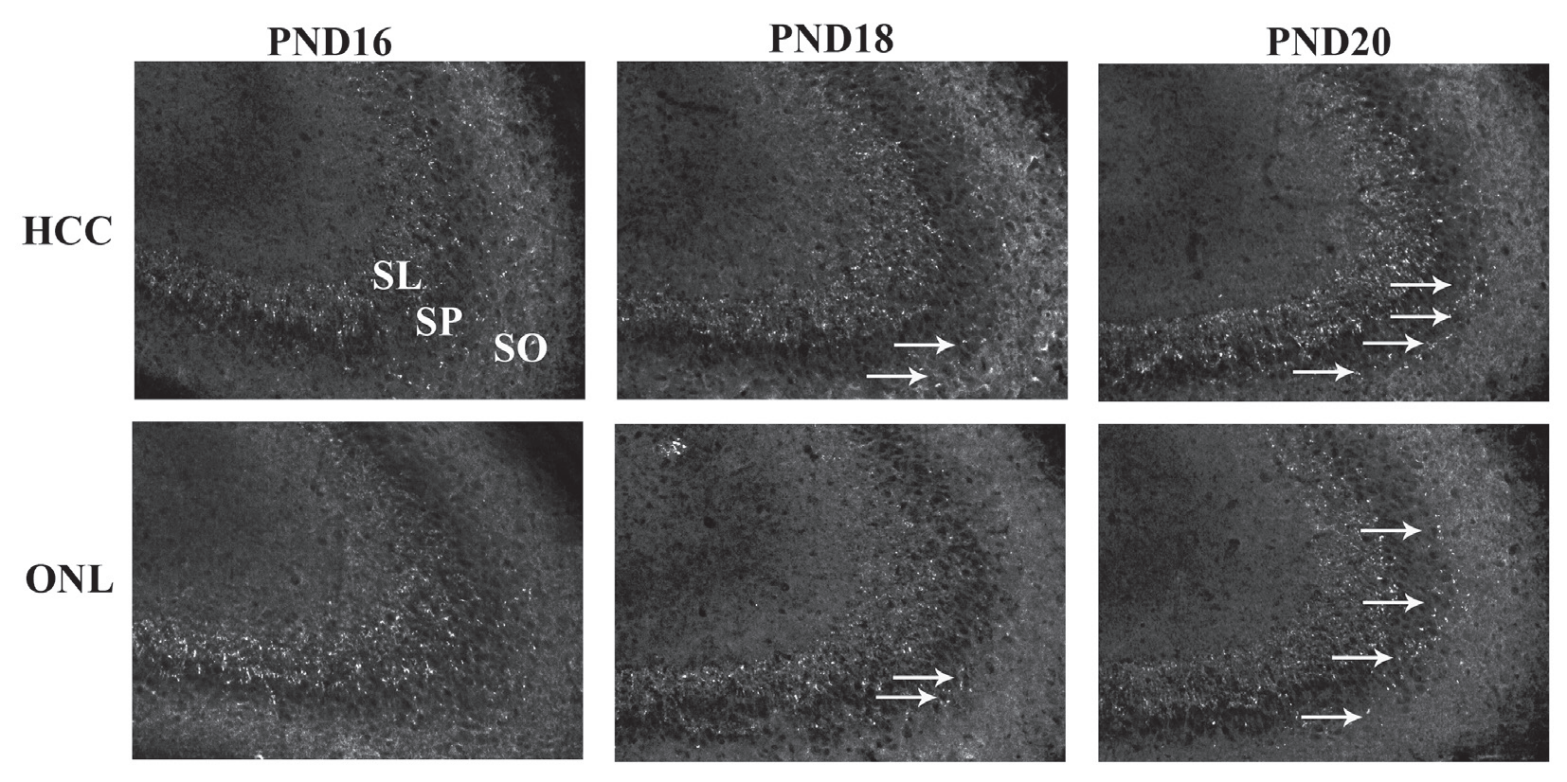
Synaptophysin staining in the CA3 region as an indicator of mossy fiber (MF) projections. Brains from PND16, PND18 and PND20 from home cage control (HCC) and object in a novel location (ONL) groups were removed and processed immunohistochemically for synaptophysin staining in the CA3 region as a marker for MF connectivity. Top panel shows representative staining from the three age groups from the HCC condition and bottom panel show staining from the ONL condition. Abbreviations: SO: stratum oriens; SP: stratum pyramisale; SL: stratum lucidum. Arrows point to synaptophysin-positive puncta in the SO region.

***Stratum Lucidum (SL).*** The average number of synaptophysin-positive puncta ( Dataset 3) in the SL ( Figure 3A and Supplemental Figure S1) was analyzed using a 3×3 univariate ANOVA (age (PND16, PND18, PND20) and training history (HCC, ONL, MWM) as the fixed factors). This analysis revealed a main effect of age only (F(2,2) = 8.53, p < 0.01) (training history F(2,4) < 1.0; interaction F(4,42) < 1.0). Tukey HSD post-hoc comparisons on the main effect of age revealed that the PND16 group showed more synaptophysin-positive puncta in the SL than both the PND18 and PND20 groups (p < 0.05).

***Stratum Oriens (SO).*** The average number of synaptophysin-positive puncta ( Dataset 3) in the SO ( Figure 3B and Supplemental Figure S1) was analyzed using a 3×3 univariate ANOVA (age (PND16, PND18, PND20) and training history (HCC, ONL, MWM) as the fixed factors). This analysis revealed a main effect of age only (F(2,2) = 91.78, p < 0.001) (training history F(2,4) < 1.0; interaction F(4,42) < 1.0). Tukey HSD post-hoc comparisons on the main effect of age revealed that the PND18 group showed more synaptophysin-positive puncta in the SO than the PND16 group (p < 0.01) and the PND20 group showed more synaptophysin-positive puncta in the SO than both the PND16 and PND18 groups (p < 0.001; Figure 3B).

***SO:SL Ratio.*** The ratio (see Dataset 3) of synaptophysin-positive puncta in the SO to puncta in the SL ( Figure 3C and Supplemental Figure S1) was analyzed using a 3×3 univariate ANOVA (age (PND16, PND18, PND20) and training history (HCC, ONL, MWM) as the fixed factors). This analysis revealed a main effect of age only (F(2,2) = 91.52, p < 0.001) (training history F(2,4) < 1.0; interaction F(4,42) < 1.0). Tukey HSD post-hoc comparisons on the main effect of age revealed that the PND18 group showed more synaptophysin-positive puncta in the SO than the PND16 group (p < 0.001) and the PND20 group showed more synaptophysin-positive puncta in the SO than both the PND16 and PND18 groups (p < 0.001; Figure 3C).

Synaptophysin Quantification Dataset Synaptophysin data were collected as number of puncta within the stratum lucidum (SL), stratum oriens (SO) and the ratio of SO:SL as defined in the text ^80^. Click here for additional data file. Copyright: © 2015 Comba R et al. 2015 Data associated with the article are available under the terms of the Creative Commons Zero "No rights reserved" data waiver (CC0 1.0 Public domain dedication).

### c-Fos immunohistochemistry

**Figure 4.**
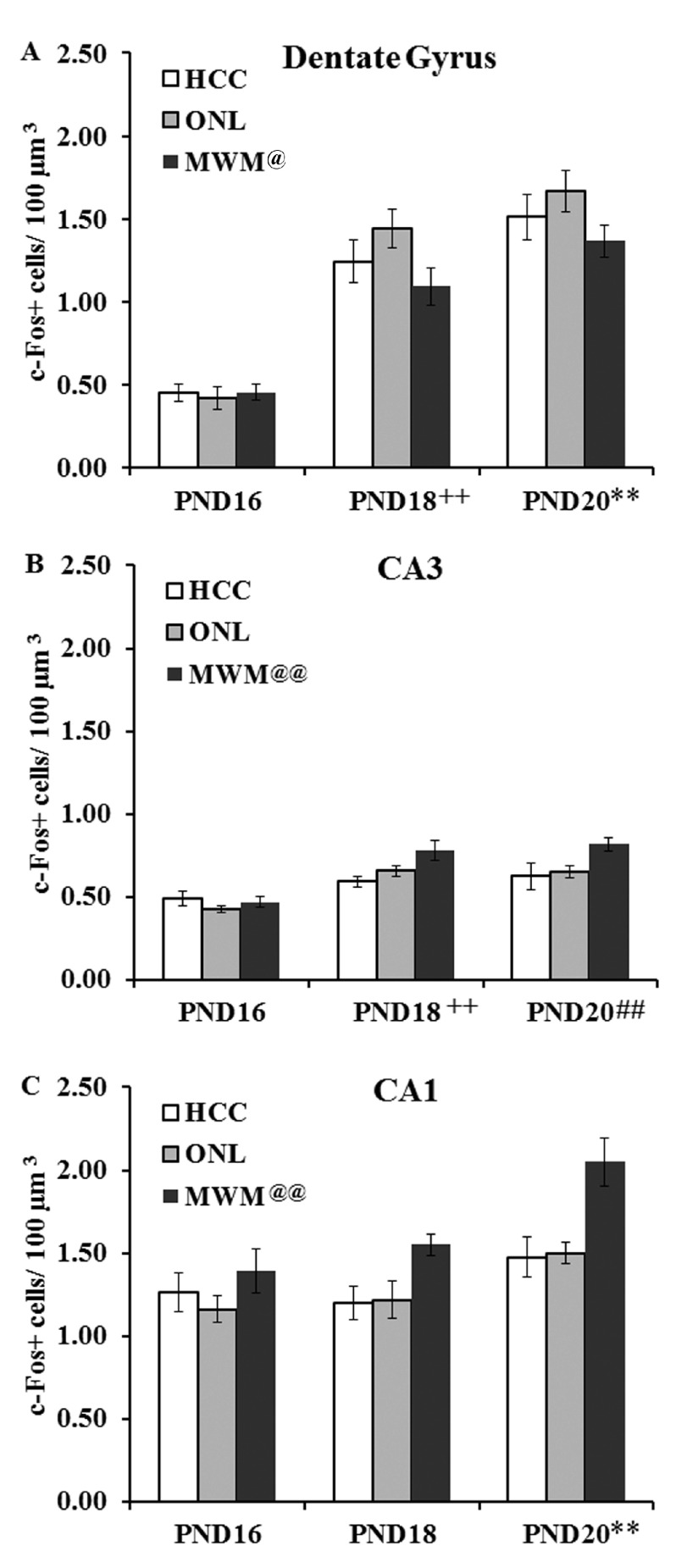
Quantification of hippocampal c-Fos labeling. The estimated number of c-Fos-positive cells per 100 µm ^3^ were quantified in the ( **A**) dentate gyrus (DG); ( **B**) CA3 and ( **C**) CA1 hippocampal regions from PND16, PND18 and PND20 groups from home cage control (HCC), object in a novel location (ONL) or Morris water maze (MWM) conditions. ( **A**) DG c-Fos staining revealed more c-Fos-positive cells in the PND18 than the PND16 group (++, p < 0.01) and more positive cells in the PND20 group than the PND16 and PND18 groups (*** p < 0.01). ( **B**) CA3 staining revealed more c-Fos positive cells in the PND18 than the PND16 group (++, p < 0.01) and in the pND20 group than the PND16 group (##, p < 0.01). There was also more c-Fos labelling associated with the MWM condition than the HCC and ONL conditions (@@, p < 0.01). ( **C**) CA1 staining revealed more c-Fos positive cells in the PND20 than the PND16 and PND18 groups (**, p < 0.01). There was also more c-Fos labelling associated with the MWM condition than the HCC and ONL conditions (@@, p < 0.01).

**Figure 5.**
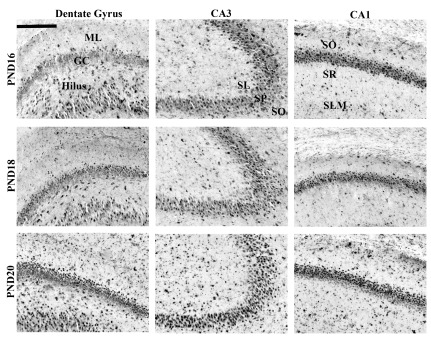
Representative c-Fos labeling in the hippocampus. Representative images for immunohistochemical localization of c-Fos in the dentate gyrus (DG), CA3 and CA1 regions. All images are from rats included in the MWM condition. Top row shows sections from PND16; middle row from PND18; bottom row from PND20. Images were taken at 10× with scale bar = 200 μm. Abbreviations: ML: molecular layer of the dentate gyrus; GC: granule cells; S: *stratum lucidum*; SP: *stratum pyramidale*; SO: *stratum oriens*; SR: *stratum radiatum*; SLM: *stratum lacunosum moleculare*.

**Figure S2.**
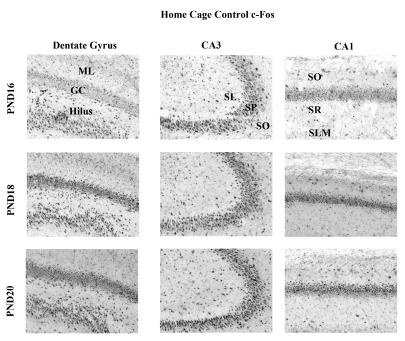
Representative c-Fos labeling in the hippocampus. Representative images for immunohistochemical localization of c-Fos in the dentate gyrus (DG), CA3 and CA1 regions from rats included in the HCC condition. Top row shows sections from PND16; middle row from PND18; bottom row from PND20. Images were taken at 10×. Abbreviations: ML: molecular layer of the dentate gyrus; GC: granule cells; S: *stratum lucidum*; SP: *stratum pyramidale*; SO: *stratum oriens*; SR: *stratum radiatum*; SLM: *stratum lacunosum moleculare*.

**Figure S3.**
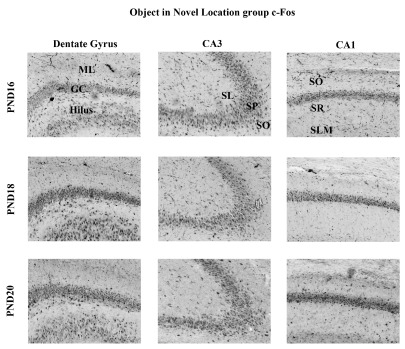
Representative c-Fos labeling in the hippocampus. Representative images for immunohistochemical localization of c-Fos in the dentate gyrus (DG), CA3 and CA1 regions from rats included in the ONL condition. Top row shows sections from PND16; middle row from PND18; bottom row from PND20. Images were taken at 10×. Abbreviations: ML: molecular layer of the dentate gyrus; GC: granule cells; S: *stratum lucidum*; SP: *stratum pyramidale*; SO: *stratum oriens*; SR: *stratum radiatum*; SLM: *stratum lacunosum moleculare*.

***Dentate Gyrus (DG).*** The estimated number of c-Fos-positive cells per 100 µm ^3^ (see Dataset 4) in the DG (quantified in Figure 4A; representative images from rats trained on the MWM shown in Figure 5 and for groups HCC and ONL in Supplemental Figure S2 and Supplemental Figure S3) was analyzed using a 3×3 univariate ANOVA (age (PND16, PND18, PND20) and training history (HCC, ONL, MWM) as the fixed factors). This analysis revealed main effects of age (F(2,2) = 96.99, p < 0.001) and training history (F(2,4) = 3.45, p < 0.05) but no significant interaction (F(4,42) = 1.23). Tukey HSD post-hoc comparisons on the main effect of age revealed that the PND18 group showed more c-Fos positive cells in the DG than the PND16 group (p < 0.01) and the PND20 group showed more c-Fos positive cells in the DG than both other age groups (p < 0.001; Figure 4A). Post-hoc analysis of the main effect of training history revealed fewer c-Fos positive cells associated with MWM task than ONL task (p < 0.05).

***CA3.*** The estimated number of c-Fos-positive cells per 100 µm ^3^ (see Dataset 4) in the CA3 (quantified in Figure 4B; representative images from rats trained on the MWM shown in Figure 5 and for groups HCC and ONL in Supplemental Figure S2 and Supplemental Figure S3) was analyzed using a 3×3 univariate ANOVA (age (PND16, PND18, PND20) and training history (HCC, ONL, MWM) as the fixed factors). This analysis revealed main effects of age (F(2,2) = 28.43, p < 0.001) and training history (F(2,4) = 7.08, p < 0.01) but no significant interaction (F(4,42) = 1.95). Tukey HSD post-hoc comparisons on the main effect of age revealed that the PND20 and PND18 groups showed more c-Fos positive cells in the CA3 region than the PND16 group (p < 0.001). Post-hoc analysis of the main effect of training history revealed more c-Fos staining associated with the MWM task than the ONL task and the HCC condition (p < 0.01 for both comparisons).

***CA1.*** The estimated number of c-Fos-positive cells per 100 µm ^3^ (see Dataset 4) in the CA1 (quantified in Figure 4C; representative images from rats trained on the MWM shown in Figure 5 and for groups HCC and ONL in Supplemental Figure S2 and Supplemental Figure S3) was analyzed using a 3×3 univariate ANOVA (age (PND16, PND18, PND20) and training history (HCC, ONL, MWM) as the fixed factors). This analysis revealed main effects of age (F(2,2) = 14.21, p < 0.001) and training history (F(2,4) = 12.49, p < 0.001) but no significant interaction (F(4,42) = 1.26). Tukey HSD post-hoc comparisons on the main effect of age revealed that the PND20 group showed more c-Fos positive cells in the CA1 region than both other age groups (p < 0.001). Post-hoc analysis of the main effect of training history revealed more c-Fos staining associated with the MWM task than the ONL task and the HCC condition (p < 0.01 for both comparisons).

C-Fos Quantification Dataset Stereological summary of the number of c-Fos positive cells within the three hippocampal regions (dentate gyrus (DG), CA1 and CA3) for the three behavioral conditions. Data are expressed as mean number of c-Fos-positive cells per 100 µm ^3^ ^81^. Click here for additional data file. Copyright: © 2015 Comba R et al. 2015 Data associated with the article are available under the terms of the Creative Commons Zero "No rights reserved" data waiver (CC0 1.0 Public domain dedication).

## Discussion

### Summary of main findings

 The purpose of the current work was three-fold: 1) to determine whether the emergence of spatial-behavioral function was based on the ability to generate appropriate behavioral output; 2) to assess whether mossy fiber connectivity patterns preceded optimal spatial-behavioral function; 3) to explore functional changes in the hippocampus to determine whether activity in hippocampal networks occurred in a training-dependent or developmentally-dependent fashion. At PND20, optimal spatial behavioral performance emerged on both the water maze task and the object exploration task whereas at PND16 and PND18, optimal spatial behavior was not present on either task. Analysis of synaptophysin staining in the CA3 SL and SO subregions revealed more synaptophysin staining in the SO in the PND18 than the PND16 group and more synaptophysin staining in the SO in the PND20 group than both the PND16 and PND18 groups. Estimates of c-Fos-positive cells in the DG, CA3 and CA1 regions revealed more staining in the DG and CA3 regions in the PND18 and PND20 groups than the PND16 group (and more in the PND20 versus PND18 group) as well as more c-Fos staining in the CA3 and CA1 areas associated with water maze training. The combined behavioral and immunohistochemical results support the hypothesis that hippocampal anatomical and functional neural substrates mature prior to the emergence of optimal spatial-behavioral function. The maturation of these networks may be necessary to support the emergence of optimal spatial behavioral performance.

### Ontogeny of spatial behavior: performance or cognition?

Training on two tasks (water and dry maze) that required distinct behavioral topology (swimming versus walking) to complete were used. In this case, the PND20 group showed evidence of optimal spatial proficiency on both tasks when compared to the PND16 and PND18 groups. Behavioral results revealed that the PND20 group given 1 day of water maze training had significantly lower latencies than the groups trained at PND16 or PND18 suggesting the emergence of spatial function by PND20. The PND20 group also showed significantly faster swim speeds. This raises the possibility that the older rats were better able to perform the task than PND16 and PND18 rats because they were stronger and possibly, better swimmers. Rather than spatial function being the reason for the superior water maze performance in the PND20 group, it could be argued that physicality contributed to superior water maze task performance. Because swimming requires a certain level of physical aptitude, an older rat may show better performance because they have more muscle mass to perform the task.

To examine whether the improvements seen at PND20 were due to spatial or motor development, an experiment was run on a dry maze. This task was less physically demanding than the water maze task (ambulation rather than swimming) and was hypothesized to tease out spatial from motor development. Other reports have shown that by PND15/PND16, rats are able to perform tests of locomotor activity and engage in exploratory behaviours such as rearing that are similar to adults ^8, 74^ and what was required for performance of the ONL task used in the present study. In contrast, optimal (i.e., adult) swimming behavior does not fully emerge until PND22 ^68^ though PND15 rats are able to keep their face and nose out of water. Thus, performance on the ONL task was utilized as a less physically demanding task yet still able to tap into spatial functions. For the ONL task, the PND20 group performed significantly better than chance but the younger groups (PND16 and PND18) showed chance performance. Because the ONL task takes advantage of the ability to detect changes in the spatial relationship between the two objects but is less physically demanding than the water maze, a preliminary conclusion is that optimal spatial behavioral function emerges around PND20, independent from behavioral topology.

Converging lines of evidence from other behavioral studies point to the postnatal period from PND16 to PND21 as a sensitive developmental timeframe for spatial behavioral function to emerge. Improved performance on a number of spatial tasks during development ^2– 5, 13, 16, 20, 21, 29, 32, 38, 41, 46, 64– 66, 70, 71, 73, 76^ point to a sensitive developmental period for spatial behavior to emerge between PND16 and PND21. In addition, optimal performance on a delayed alternation task has been shown to emerge between PND19 and PND27 but in pups with PND10 hippocampal lesions, this behavior fails to fully develop ^23^ suggesting that hippocampal integrity is important for the normal development of spatial behavioral function and other brain structures do not compensate when the hippocampus is damaged during postnatal development ^6^.

### Ontogeny of Mossy Fiber Connectivity

In adult Long Evans rats (LER), the mossy fiber terminal field (as labeled with zinc or synaptophysin) shows a dense projection to the *stratum oriens* (SO) corresponding to the basal dendrites of the CA3 pyramidal cells ^33^. In an examination of the development of CA3 hippocampal mossy fiber (MF) distribution in LER ^32^, MF innervation of the *stratum lucidum* (SL) was widespread by PND12 and beginning on PND15, MF staining was evident in the *stratum pyramidale* (SP) and by PND18 and PND21, widespread MF staining was observed in the SO. By PND24, the SO projection in LER was complete and remained stable into adulthood ^33^.

The present study carried out an explicit comparison between the development of these MF connectivity patterns and the emergence of spatial behavior. While in a previous study ^41^, using LER rats, dramatic improvement in spatial behavioral function was observed from PND18 to PND21, there was no examination of the MF distribution during this course of behavioral improvement. Utilizing a one-day training procedure on two different tasks and comparing the change in MF connectivity patterns to a home cage control condition allowed us to determine how plasticity in the MF terminal field was associated with the emergence of spatial behavior. In this respect, clear developmental-dependent changes occurred in MF terminal field distribution (inferred from synaptophysin staining) with a gradual increase in staining observed in the SO from PND16 to PND18 to PND20 with SO staining being greatest at PND20. This suggests that developmentally-dependent changes in the distribution of MF terminals, particularly those that terminate on the basal dendrites of CA3 pyramidal neurons, may form an anatomical substrate that supports the emergence of spatial behavior. Such facilitation of spatial behavior could arise as a consequence of shifting input away from inhibitory interneurons ^10, 51, 53^ thereby reducing the number of synapses on inhibitory interneurons located within the SL ^72^ leading to excitatory inputs on CA3 pyramidal neurons via the basal dendrites in SO.

### Ontogeny of hippocampal network activity

The developmentally-dependent changes in presynaptic MF terminals are likely dependent on patterned neural activity within this system that occurs during development ^39^. To examine putative functional changes associated with developmentally-linked improvements in spatial behavior and MF terminal field distribution, c-Fos immunohistochemical staining in the DG, CA3 and CA1 regions of the hippocampus was carried out across the three developmental time points (PND16, PND18 and PND20). Immediate early gene proteins, such as c-Fos, regulate the transcription of additional genes directing a general genomic response to a variety of environmental stimuli ^69^. These regulatory proteins control downstream gene expression and are thought to translate environmental signals into relatively long-term changes in neuronal function ^28, 40, 69^. As such, c-Fos protein labeling was used in the present work to provide a marker for cells that have recently been activated and are potentially undergoing long-term structural or functional changes.

Comparison of c-Fos-positive cells between the different ages revealed more c-Fos labeled neurons in the DG and CA1 regions in the PND20 group than the PND16 and PND18 groups indicating more network activity in these regions at PND20 that may facilitate the emergence of spatial behavior. The PND18 group also showed more c-Fos labeling in the DG and CA3 than the PND16 group, perhaps providing the patterned neural activity required for development changes in the MF connectivity. Finally, there were training-associated elevations in c-Fos staining following the water maze task in the CA3 and CA1 regions.

The developmental-associated changes in c-Fos labeling in the DG and CA1 regions continued to show increased activity from PND16 to PND18 then more activity at PND20 while the CA3 labeling patterns suggested a plateau of activity at PND20. Activity changes in the DG during development may reflect patterned activity necessary for the stabilization of MF inputs to CA3. Indeed, the wave of increased DG activity at PND18 corresponds to the first occurrence of a significant MF projection to SO; the second wave of DG activity at PND20 corresponds to a further, significant expansion of the MF projection within SO. With the formation and stabilization of this MF-CA3 network, optimal spatial behavior may be possible. A previous report showed that the expression of Homer1a in the DG peaks between PND19 and PND23 ^52^ compared to PND9-15 and PND35 to adulthood. This is consistent with the current results showing a continued maturation of network activity in the DG spanning the PND16 – PND20 period. During this maturational period, appropriate networks may be established to support optimal spatial behavioral output.

The CA1 region also showed continued maturation of network activity spanning PND16 – PND20 (with c-Fos-positive neurons at PND18 > PND16 and PND20 > PND18). Activity patterns in the CA1 region may continue to mature well beyond the PND20 period. As an example, place cells in CA1 with specific spatial firing patterns have been recorded at PND17 with place cell patterns conveying optimal spatial information showing continued development up to PND35 ^1, 46^. Likewise, place cell firing is present from PND16 – PND26 but continues to improve throughout development with stable place cell recordings (i.e., similar to adults) being made at PND28 ^76^. Theta-modulated firing is present at PND16 in the hippocampus CA1 region and these responses reach adult proportions by PND22 ^46, 76^. After PND21, both the magnitude and threshold for post-synaptic induction of long-term potentiation (LTP) are reduced with a corresponding increase in the threshold for presynaptic induction ^13, 22^. These data, with the present results, suggest continued maturation of CA1 neural activity beyond the PND20 time point that continues to support the refinement and optimization of spatial information.

The CA3 and CA1 subregions showed patterned c-Fos labeling that was associated with the water maze task condition. While studies have shown that the dorsal CA3 and CA1 hippocampal subregions contribute to numerous aspects of spatial processing ^31, 36, 37, 45^, there are functional differences between the two regions. The CA3 subregion has been suggested to be important for cognitive functions associated with spatial pattern separation ^44, 63^, spatial pattern completion ^48^, novelty detection ^47^ and short-term memory ^45, 49^. Likewise, the dorsal CA3 region has been shown to be involved in the early stages of acquiring spatial information but less involved during the retrieval of spatial information after long delays ^50^. The dorsal CA1 region has been shown to be involved in the encoding and retrieval of spatial-shock associations ^35^ and inactivation of the CA1 region with lidocaine prior to water maze training impairs spatial performance ^14, 55, 61^. As well, CA1 lidocaine injections before a probe retention test impaired the ability of rats to demonstrate spatial function ^15, 56^.

In the present work, both the CA3 and CA1 regions showed c-Fos elevations associated with performance of the water maze task. Because there was no probe retention test, this patterned activity appears to be related to the acquisition of spatial information. For the ONL task, the animals were euthanized following the test session. In this case, there was no significant increase in c-Fos labeling over and above the HCC condition. This suggests the possibility that these regions were not activated during the ONL task or that they were not involved in the retrieval of spatial information. While the present study was not designed to examine differential activation patterns in the CA3 and CA1 regions based on different cognitive demands, it would be of interest to determine if distinct mnemonic functions (e.g., pattern separation mediated by the DG-CA3 network ^42, 62^ and pattern completion mediated by the CA3-CA1 network ^43^) would show a different pattern of emergence during development.

## Conclusions

The combination of neuroanatomical and behavioural results from the present work leads to the hypothesis that this developmental time period (PND16 – PND20) represents a sensitive period for hippocampal anatomical and functional modification leading to the emergence of spatial behavior. The main purpose of the present study was to investigate the relationship between the emergence of spatial-behavior function and the emergence of the neural substrates – both anatomical and functional – that would support optimal spatial-behavioral function. In this respect, MF connectivity-based developmental changes preceded the emergence of spatial behavior. In association with this, functional aspects of hippocampal neurons changed in response to developmental age with functional changes also occurring in response to behavioral-dependent inputs. These data support the hypothesis that network maturation the hippocampus supports the emergence of optimal spatial behavior.

## Data availability

The data referenced by this article are under copyright with the following copyright statement: Copyright: © 2015 Comba R et al.

Data associated with the article are available under the terms of the Creative Commons Zero "No rights reserved" data waiver (CC0 1.0 Public domain dedication). 

*F1000Research*: Dataset 1. Watermaze Dataset, 10.5256/f1000research.6822.d96530 ^78^

*F1000Research*: Dataset 2. Drymaze Dataset, 10.5256/f1000research.6822.d96531 ^79^

*F1000Research*: Dataset 3. Synaptophysin Quantification Dataset, 10.5256/f1000research.6822.d96532 ^80^

*F1000Research*: Dataset 4. C-Fos Quantification Dataset, 10.5256/f1000research.6822.d96533 ^81^
