# Supplementary material for: Emergence of spatial behavioral function and associated mossy fiber connectivity and c-Fos labeling patterns in the hippocampus of rats
Source: F1000Res. 2015 Jul 27;4:396. [Version 1] doi: 10.12688/f1000research.6822.1 (PMC4712777; doi:10.12688/f1000research.6822.1)
Supplement: Peer review history

# Peer review: Referee response for version 1

**DOI:** 10.5256/f1000research.7335.r9641

**Published:** 2015-08-20

**Authors:** Bryan D. Devan, Christoper Magalis, Robert McDonald

**Affiliations:** 1 Laboratory of Comparative Neuropsychology, Psychology Department, Towson University, Towson, MD, USA; 2 Psychology Department, Towson University, Towson, MD, USA; 3 Canadian Centre for Behavioural Neuroscience, Department of Neuroscience, University of Lethbridge, Lethbridge, AB, Canada

**Editorial Note from F1000Research – 26 ^th^ August 2015:**

****

**This report has been updated to provide a link to the author's now published Correspondence Article. The phrase "(forthcoming)"** **was removed**. **No other changes have been made to the text of the report.**

The study by Comba *et al*. ^1^ suggests a critical period of prenatal development (PND) in rodents during which neuronal mossy fiber growth in the hippocampus is associated with the emergence of spatial behavior. The researchers emphasize the PND 15-18 month period where this growth connecting the dentate gyrus to the CA3 cellular field is most prominent. It is also noted in the introduction that such grow may be related to the finding that neurogenesis-based processes specific to the hippocampus are also associated with the emergence of spatial learning. The researchers describe a “behavioral typology” control in their research design to dissociate effects based on non-cognitive motor demands from true cognitive information processing, which was supported by their data analyses of place learning in the Morris water maze versus spatial exploration in a dry land task (with swimming being more difficult than common ambulation). Despite the difference in motor demands, there was a common emergence of spatial behavior proficiency on each task at PND20. The researchers also found that enhanced mossy fiber projections, revealed by synaptophysin staining in the CA3 region, preceded the emergence of spatial behavior. Developmentally-dependent functional changes in cFOS positive cells were increased in all hippocampal subregions measured, while training-dependent changes were restricted to the CA3 and CA1 regions for groups trained in the water maze. The researchers conclude that mossy fiber connectivity along with enhanced function of the hippocampus precedes the emergence of spatial behavior at PND20, confirming their hypothesis of a sensitive period for hippocampal growth and the emergence of cognitive-spatial function.

This research is very important and exciting when considering past studies of place learning ability in adult rats and research attempts to dissociate motor performance from true cognitive processing.  In previous water maze studies of latent learning ^2^^,^^3^ using passive placement on the goal in the water maze to view distal cues to form a cognitive map of the environment, mixed results and individual differences seem to obscure matters ^4^^-^^8^, leading to one interpretation that movement and cognitive mapping may necessarily occur simultaneously, a finding that has been replicated in humans using a virtual version of the water maze ^9^. Hence, for the water maze at least, movement through the environment seems to be an important constraint on highly proficient spatial learning and navigation. The use of a separate dry land task by Comba *et al. *with less motoric demands seems to be in agreement with the difference in motor demands between the original rodent version of the task ^10^ requiring swimming and the human virtual version ^9^ using minimal hand/finger movements to navigate. The role of dynamic movement during spatial tasks and the motoric demands have been topics of intense interest with the role of the hippocampus as a substrate for cognitive mapping ^11^^,^^12^, path integration ^13^^-^^17^, or the conductor of a symphony of dynamic movement and mapping ^18^ as part of a larger neural network of brain systems ^19^ have all been hotly debated theoretically over the years. The separation of cognitive and motor performance associated with developmentally-specific changes in hippocampal circuitry by Comba *et al*. is an exciting finding that may have important implications for a central role of the hippocampus in cognitive-spatial information processing as it emerges early in development.

Consequently, the “behavioral typology” issue in the Comba *et al*. study, along with other aspects of their research design, is of critical importance in assessing the emerging role of the hippocampus in cognitive-spatial behavior. The following matters should be considered by all interested in this fascinating area of research in general, and in the Comba *et al*. study in particular.

**Specific Considerations**

1. The level of spatial proficiency in escaping to a hidden platform for PND20 rats given only 8 trials in the water maze is not comparable to the asymptotic level of escape latency performance (< 10 sec) observed in most water maze studies after considerably more extensive training.  The one day water maze training paradigm is likely tapping into ventral hippocampal function in which the animals are just approaching the general location. After more training, dorsal hippocampus forms a more precise representation of the location. The authors should discuss this work ^20^ and an analysis of their data (dorsal versus ventral) would be of interest.
2. On a related matter, escape is not required, and exploration of an object at a novel “place” in the dry land task is very different from the typical water maze procedure, involving the presence of a local cue or familiar beacon (with different motivation). Some might argue that the lack of “true” spatial proficiency in the water maze is a flaw or weakness of the study; however, given the focus on the emergence of spatial behavior, and that well-learned escape responses are dependent on other brain regions ^21^^,^^22^ that contribute/correlate with movement parameters ^19^, it seems reasonable to expect less performance-wise using the escape latency measure than what is typically observed in most studies of this type.
3. Consequently, spatial bias on a probe test might be considered as an alternative measure in future studies as it does not depend on a well-learned escape response that may be less hippocampal-dependent and more closely approximates the dwell time that is measured on the spatial exploration task.
4. The object/place task is interesting. Integration with ideas about direct versus indirect measures of memory and the role of the hippocampus in one versus the other, and how these ideas relate to their different measures on this task would be of interest ^23^.
5. There are other obvious differences between the two tasks. Water maze for example is not disrupted by disorientation procedures but a dry-land version of spatial localization is disrupted by disorientation ^24^. This work suggests that the representations are different as well, not just the behavioral topology. This should be discussed.
6. The number of rats in each group for the water maze task is quite low (n = 5/group).
7. A statement on the standardization of immunohistochemical procedures would be reassuring for those not familiar with the specific techniques used. Also, the use of an unbiased stereology technique should be considered.
8. It appears that different behavioral procedures may have been conducted at different institutions. A statement on the time of day of testing and other procedural controls would provide reassurance that there are no threats to internal validity.
9. More information on the recording and quantification of exploratory behavior (e.g., video recording, tracking, and interrater reliability) would be helpful for assessment and replication.
10. It is interesting that the researchers note that PND18 rodents traveled a longer distance in the novel relocation task then the other groups (even though apparently PND20 rats exhibited more exploratory behavior). This finding may warrant further discussion to support the argument that both tasks assess potentially related cognitive functions.
11. Reassurance that no statistical assumptions were violated (e.g., spherecity). Tukey HSDs are specifically based on studentized q related statistics but t-tests were reported. Though this may have been simply an alternative method (e.g. regression-based) to report the Tukey post-hoc results, possibly Bonferroni t-tests with separate mean square error denominators may be optimal for potential corrections to assumption violations.

See full Correspondence Article by the Referees

We have read this submission. We believe that we have an appropriate level of expertise to confirm that it is of an acceptable scientific standard.

## References

[1] CombaRGervaisNJMumbyDGHolahanMR: Emergence of spatial behavioral function and associated mossy fiber connectivity and c-Fos labeling patterns in the hippocampus of rats [v1; ref status: approved 1, http://f1000r.es/5nr]. *F1000Research*.2015;4(396) : 10.12688/f1000research.6822.1 10.12688/f1000research.6822.1 PMC471277726925223

[2] KeithJRMcVetyKM: Latent place learning in a novel environment and the influences of prior training in rats. *Psychobiology*.1988;16(2) : 10.3758/BF03333116 146-151 10.3758/BF03333116 Reference source

[3] SutherlandRJLinggardR: Being there: a novel demonstration of latent spatial learning in the rat. *Behav Neural Biol.*.1982;36(2) : 10.1016/S0163-1047(82)90101-7 103-107 10.1016/S0163-1047(82)90101-7 Reference source 7183307

[4] DevanBDPetriHLMishkinMStoufferEMBowkerJLYinPBBuffalariDMOldsJL: A room with a view and a polarizing cue: individual differences in the stimulus control of place navigation and passive latent learning in the water maze. *Neurobiol Learn Mem.*.2002;78(1) : 10.1006/nlme.2001.4057 79-99 10.1006/nlme.2001.4057 Reference source 12071669

[5] ChewGLSutherlandRJWhishawIQ: Latent learning does not produce instantaneous transfer of place navigation: A rejoinder to Keith and McVety. *Psychobiology*.1989;17(2) : 10.3758/BF03337838 207-209 10.3758/BF03337838 Reference source

[6] WhishawIQ: Latent learning in a swimming pool place task by rats: Evidence for the use of associative and not cognitive mapping processes. *Q J Exp Psychol B.*.1991;43(1) : 10.1080/14640749108401260 83-103 10.1080/14640749108401260 Reference source 2017576

[7] JacobsWJZaborowskiJAWhishawIQ: Failure to find latent spatial learning in the Morris Water Task: Retraction of Jacobs, Zaborowski, and Whishaw. *Journal of Experimental Psychology: Animal Behavior Processes*.1989;15(3) : 10.1037/0097-7403.15.3.286 286 10.1037/0097-7403.15.3.286 Reference source

[8] JacobsWJZaborowskiJAWhishawIQ: Jacobs/WJ;Zaborowski/JA;Whishaw/IQ. *Journal of Experimental Psychology: Animal Behavior Processes*.1989;15(1) :36-42 Reference source

[9] HamiltonDADriscollISutherlandRJ: Human place learning in a virtual Morris water task: some important constraints on the flexibility of place navigation. *Behav Brain Res.*.2002;129(1-2) : 10.1016/S0166-4328(01)00343-6 159-170 10.1016/S0166-4328(01)00343-6 Reference source 11809507

[10] MorrisRGM: Spatial localization does not require the presence of local cues. *Learning and Motivation*.1981;12(2) : 10.1016/0023-9690(81)90020-5 239-260 10.1016/0023-9690(81)90020-5 Reference source

[11] O'KeefeJNadelL: The hippocampus as a cognitive map. *Oxford University Press*.1978; Reference source

[12] TolmanEC: Cognitive maps in rats and men. *Psychol Rev.*.1948;55(4) : 10.1037/h0061626 189-208 10.1037/h0061626 Reference source 18870876

[13] WhishawIQJarrardLE: Evidence for extrahippocampal involvement in place learning and hippocampal involvement in path integration. *Hippocampus.*.1996;6(5) : 10.1002/(SICI)1098-1063(1996)6:5<513::AID-HIPO4>3.0.CO;2-J 513-524 10.1002/(SICI)1098-1063(1996)6:5<513::AID-HIPO4>3.0.CO;2-J Reference source 8953304

[14] WhishawIQMaaswinkelH: Rats with fimbria-fornix lesions are impaired in path integration: a role for the hippocampus in "sense of direction". *J Neurosci.*.1998;18(8) :3050-3058 Reference source 952602210.1523/JNEUROSCI.18-08-03050.1998PMC6792581

[15] WhishawIQ: Place learning in hippocampal rats and the path integration hypothesis. *Neurosci Biobehav Rev.*.1998;22(2) : 10.1016/S0149-7634(97)00002-X 209-220 10.1016/S0149-7634(97)00002-X Reference source 9579312

[16] WhishawIQMcKennaJEMaaswinkelH: Hippocampal lesions and path integration. *Curr Opin Neurobiol.*.1997;7(2) : 10.1016/S0959-4388(97)80011-6 228-234 10.1016/S0959-4388(97)80011-6 Reference source 9142750

[17] WhishawIQCasselJCJarradLE: Rats with fimbria-fornix lesions display a place response in a swimming pool: a dissociation between getting there and knowing where. *J Neurosci.*.1995;15(8) :5779-5788 Reference source 764321810.1523/JNEUROSCI.15-08-05779.1995PMC6577651

[18] The navigating hippocampus: An individual medley of movement, space, and memory. In: Buzsáki G and Vanderwolf CHV(eds): Electrical activity of the archicortex. *Akadémiai Kiadó*.1985;255-279

[19] MaguireEABurgessNDonnettJGFrackowiakRSFrithCDO'KeefeJ: Knowing where and getting there: a human navigation network. *Sciece.*.1998;580(5365) : 10.1126/science.280.5365.921 921-924 10.1126/science.280.5365.921 Reference source 9572740

[20] RuedigerSSpirigDDonatoFCaroniP: Goal-oriented searching mediated by ventral hippocampus early in trial-and-error learning. *Nat Neurosci.*.2012;15(11) : 10.1038/nn.3224 1563-1571 10.1038/nn.3224 Reference source 23001061

[21] DevanBDGoadEHPetriHL: Dissociation of hippocampal and striatal contributions to spatial navigation in the water maze. *Neurobiol Learn Mem.*.1996;66(3) : 10.1006/nlme.1996.0072 305-323 10.1006/nlme.1996.0072 Reference source 8946424

[22] SutherlandRJRodriguezAJ: The role of the fornix/fimbria and some related subcortical structures in place learning and memory. *Behav Brain Res.*.1989;32(3) : 10.1016/S0166-4328(89)80059-2 265-277 10.1016/S0166-4328(89)80059-2 Reference source 2496702

[23] MosesSNSutherlandRJMcDonaldRJ: Differential involvement of amygdala and hippocampus in responding to novel objects and contexts. *Brain Res Bull.*.2002;58(5) : 10.1016/S0361-9230(02)00820-1 517-527 10.1016/S0361-9230(02)00820-1 Reference source 12242105

[24] GibsonBMShettleworthSJMcDonaldRJ: Finding a goal on dry land and in the water: differential effects of disorientation on spatial learning. *Behav Brain Res.*.2001;123(1) : 10.1016/S0166-4328(01)00196-6 103-111 10.1016/S0166-4328(01)00196-6 Reference source 11377733

# Peer review: Referee response for version 1

**DOI:** 10.5256/f1000research.7335.r9642

**Published:** 2015-07-31

**Authors:** Eric M. Stouffer

**Affiliations:** 1 Department of Psychology, Bloomsburg University of Pennsylvania, Bloomsburg, PA, USA

The authors conducted behavioral and neuroanatomical experiments to determine if hippocampal neuroanatomical development precedes the development of spatial learning abilities in an early postnatal period in male rats. They examined spatial learning abilities using the Morris water maze (MWM) task and a dry-version of the object in a novel location (ONL) task at postnatal days (PND) 16, 18, and 20 using separate groups of rats. Following performance on these tasks, the rats’ hippocampi were examined using synaptophysin staining and c-Fos staining to demonstrate neuroanatomical development compared to home-cage control groups of rats. Based on the results of the study, the authors suggest that neuroanatomical development of the hippocampus is present at PND 18 compared to PND 16, but that spatial learning abilities are not expressed until PND 20. Therefore, the authors state that hippocampal neuroanatomical development does indeed precede the development of spatial learning abilities in male rats.

While I agree with the authors’ main conclusion for this study, I believe that there are some issues with the behavioral data that do not fully support other assertions made by the authors. For example, the authors conclude that the male rats showed spatial learning abilities at PND 20 in both the MWM task and the ONL task. In the MWM task, the authors point to the significant difference in escape latency between the PND 20 rats and the other two age groups. However, this difference can be explained as a result of a difference in swim speed (a physical development issue) rather than as a result of spatial learning. In fact, the authors do point this out in the discussion section as a “possibility”. I would argue that the physical development (swim speed) explanation for the difference in escape latency is not only a possibility, but rather is the most likely explanation of the significant difference in escape latency, especially given the non-difference in pathlength (arguably a more sensitive measure of spatial learning than escape latency) between the three age groups. Therefore, I do not think the authors can claim that the PND 20 rats showed spatial learning abilities in the MWM task. Perhaps if the authors had tested rats at PND 22 or PND 24 they may have seen stronger evidence of spatial learning in the MWM task.

Regarding the ONL task, the authors used two main measures of spatial learning: the investigation ratio for the displaced object compared to the non-displaced object and the investigation time for the displaced and non-displaced objects. The investigation ratio data do support the conclusion that PND 20 rats show spatial learning compared to PND 16 and PND 18 rats. The investigation time data, however, did not show significant age differences. However, careful examination of Figure 2B leads me to believe that with a larger sample size that the PND 20 rats would demonstrate a significant increase in investigation time of the displaced compared to the non-displaced objects.

Therefore, I do believe that PND 20 rats do show some evidence of spatial learning compared to PND 16 and PND 18 rats, just not as strong as the authors claim. This, however, does not detract from the fact that the authors do clearly demonstrate neuroanatomical development of the hippocampus prior to the development of spatial learning skills. That was the main point of the study, and was the authors’ main conclusion of the study. Overall, I do believe that this study does add to the existing literature.

I have read this submission. I believe that I have an appropriate level of expertise to confirm that it is of an acceptable scientific standard.
